# Implementation of Flexible Work Arrangement among Healthcare Workers in Miri Hospital—Assessment of the Validity and Reliability of Flexible Work Arrangement Perceived Benefits and Barriers Scale, and the Exploratory Study

**DOI:** 10.21315/mjms2022.29.6.9

**Published:** 2022-12-22

**Authors:** Tian Er Poh, Shirlie Chai, Yee Pin Lim, Chia Yee Wong, Francis Eng Kai Heng, Jack Siew Yu Wong

**Affiliations:** 1 Miri Hospital, Ministry of Health Malaysia, Sarawak, Malaysia; 2 Institute for Clinical Research Malaysia, Ministry of Health Malaysia, Selangor, Malaysia; 3 Clinical Research Center Miri, Ministry of Health Malaysia, Sarawak, Malaysia

**Keywords:** COVID-19, pandemic, healthcare workers, shift work, government administration

## Abstract

**Background:**

Flexible work arrangements (FWAs) have been widely implemented during the COVID-19 pandemic. We aimed to assess the validity and reliability of the FWA perceived benefits and barriers (FWAPB) scale and subsequently, to determine the preference and perceived feasibility, perceived benefits and barriers, and readiness to implement FWA among healthcare workers.

**Methods:**

We conducted a cross-sectional study using a self-administered questionnaire in Miri Hospital. The questionnaire was administered via a web survey design (Google Forms). The convenience sampling method was applied to recruit respondents. All healthcare workers in Miri Hospital who could read and understand English were invited to participate in the study. Response process validation, exploratory factor analysis, reliability analyses and descriptive statistics were performed.

**Results:**

A total of 339 respondents participated. All items had satisfactory response process indices. Exploratory factor analysis revealed a three-factor structure. Items of ‘perceived benefits-workplace management’, ‘perceived benefits-family life balance’ and ‘perceived barriers’ have high internal consistency reliability (Cronbach’s alpha = 0.852–0.884) and factor loadings. Flextime is preferred and perceived to be the most feasible work arrangement. Most agreed that FWA helps in improving social distancing among colleagues (mean = 3.65, standard deviation [SD] = 0.99) and reduces their exposure to COVID-19 (mean = 3.60, SD = 1.06). A total of 44.0% of the respondents agreed Miri Hospital is ready to implement FWA.

**Conclusion:**

The FWAPB is valid and reliable. Almost half of the respondents were positive towards the implementation of FWA. These findings contribute to the understanding of FWA, and thus increase the readiness and acceptance of such an arrangement.

## Introduction

Flexible work arrangement (FWA), is an organisational policy and practice that allows employees to choose, at least to some extent, when or where they work or to work different hours from the traditional working hours (1), which is mutually beneficial for both employees and employers. Both parties have to reach a consensus on when, where and how employees will work to meet the needs of the company (2). There are several types of FWAs, such as telecommuting, remote work, condensed work weeks, customised work hours, part-time positions and job sharing (1, 2). Previous literature reported that FWA has the advantage of offering employees the flexibility in their work design, leading to increased organisational flexibility, better work-life balance and improved organisational performance (3).

The WHO declared COVID-19 as a pandemic on 11th March 2020. Subsequently, the Malaysian government announced the first movement control order (MCO) on 18th March 2020, followed by a conditional movement control order (CMCO) and recovery movement control order (RMCO). One of the preventive measures under the MCO is to practise physical distancing to reduce exposure and slow COVID-19’s spread. Thus, many organisations and businesses are exploring adopting FWA as a new norm to practise physical distancing.

The Malaysian government has adopted several FWA strategies among the civil service sectors, such as flexible working hours and shift work. During this pandemic, working from home (remote work) has been mooted as a new FWA mechanism. However, there has not been many studies to explore the acceptance and implementation of FWA among healthcare workers working in a hospital setting. Therefore, we aimed to: i) assess the validity and reliability of the FWA perceived benefits and barriers (FWAPB) scale; ii) explore the preference and perceived feasibility of FWA; iii) determine the perceived benefits and barriers of FWA and iv) determine the readiness to implement FWA among healthcare workers at Miri Hospital.

## Methods

### Study Design, Recruitment and Setting

We conducted a cross-sectional study between August 2020 and September 2020 at Miri Hospital, a major hospital in the northern zone of Sarawak, using a self-administered questionnaire administered via web survey design (Google Forms). The convenience sampling method was applied to recruit respondents. During the respondent recruitment period, an announcement about the study was made through various departmental communication groups. In addition, we distributed and placed the invitation notice with a QR code in all departments. All healthcare workers in Miri Hospital who could read and understand English were eligible to participate in the study. The respondents were requested to respond to each question at their convenience. Those who did not consent were excluded. To ensure the privacy of the respondents, the survey was strictly anonymous and did not ask for the identity of respondents. The choice of response would not affect their performance at work in any way.

This study was divided into two parts. Firstly, there was an assessment of the validity and reliability of the FWAPB scale, which consisted of the development of the questionnaire, response process validation and internal structure evaluation. Secondly, the assessment of the implementation of FWA was made, including the preference for and perceived feasibility of FWA, its perceived benefits and barriers, and the readiness to implement FWA among healthcare workers in Miri Hospital. Details of the methods used are elaborated in the following sections.

### Questionnaire Development

In the development stage, the first and second authors reviewed the relevant contents and information related to the topic. Subsequently, items were identified and generated based on the available guidelines, grey literature, and peer-reviewed FWA-related studies (2, 4–8) to construct a preliminary version of the questionnaire. The questionnaire was drafted and consisted of four sections.

Section A of the questionnaire assessed FWA options, in which the respondents were asked ‘In your current workplace, have you used the following types of FWA?’ The items assessing their preference and perceived feasibility of the FWA options were measured on a 5-point Likert rating scale ranging from ‘1 = most preferred’ to ‘5 = least preferred’ and ‘1 = most feasible’ to ‘5 = least feasible’, respectively. The FWA options in this study included: i) telecommuting, which refers to attending the office semi-regularly (e.g. 2 or 3 times per week), performing the job remotely for part of the time, using computers and technology; ii) remote working, which refers to not attending the office at all, performing the job entirely away from the office; iii) condensed work week, which refers to four 10-h working days to maintain a 40-h work week; iv) flextime, which refers to a schedule which involves flexible start and end times of the working day, and applies five 8-h working days to maintain a 40-h work week and v) shift work, which refers to a fixed 8-h shift schedule to maintain a 40-h work week.

Section B consisted of FWAPB items, which explored the perceived benefits and barriers of FWA among the employees. The items were developed based on the literature review and expert opinions. The 21 items pertaining to the perceived benefits and barriers were then categorised into the identified preliminary domains, namely work-family balance, work-life benefits, workplace management and COVID-19 risk management. Table 1 presents the initial items and the four proposed domains. The items included both positively and negatively worded statements and were administered in a random order to eliminate the order effect.

Section C elucidated the readiness to change. To assess this, the respondents were asked to read the following statements: ‘I will opt for FWA’; ‘I am confident I am able to adapt to FWA’; and ‘Miri Hospital is ready to implement FWA’. All items in Section B and C were framed using a 5-point response scale ranging from ‘1 = strongly disagree’ to ‘5 = strongly agree’. We reversed nine items in the scoring of the scale; higher scores indicated a positive perception on all items. Lastly, Section D contained 10 questions to gather respondents’ demographic information and their current work system.

### Data Analysis

Upon receiving the completed questionnaires, a pre-processing and data cleaning step was applied to remove unconsented responses and incomplete or invalid data. The data were considered invalid when the responses contained the exact same answer to all items. After removing all the unconsented responses and incomplete or invalid data, we performed the data analysis using Microsoft Excel and SPSS version 23.0. For the assessment of the scale validity and reliability, we reviewed the response process validity using response process validity indices, whereas for the factor structure and reliability of the perceived benefits and barriers of FWA, we used exploratory factor analysis (EFA) and reliability analysis, as the section contained latent constructs.

For the exploration of the implementation of a flexible work arrangement, descriptive statistics summarised the preference for and perceived feasibility of FWA based on their job scope, the perceived benefits and barriers of FWA, and the readiness to implement FWA among healthcare workers in Miri Hospital. Normally distributed numerical data were expressed as mean ± standard deviation (SD), whereas ordinal and categorical data were summarised as frequency and percentage. The procedures are described in the next sections.

### Response Process Validation

In this study, the items assessing perceived benefits and barriers of FWA were pre-tested among the targeted population through response process validation. Response process validity is also known as face validity (9). Response process represents one of the most important sources of validity evidence (10). The Standards for Educational and Psychological Testing describe the response process validation as a procedure that contributes to the evidence pertaining to the fit between the construct and the performance or response by the respondents (10).

The Face Validity Index (FVI) is used to assess comprehensibility and clarity of each item by raters, to estimate the response process validity (9). The researcher conducted the response process validation at Miri Hospital by asking the raters to indicate whether the individual items were easily understood and clear. In this study, we invited six healthcare workers from various departments, representing the targeted population who would be responding to this tool, to evaluate the instructions and items for clarity. The rates independently rated each item based on a Likert scale ranging from 0 (i.e. poor clarity or difficult comprehensibility) to 4 (i.e. good clarity or easy comprehensibility). We welcomed feedback and suggestions to refine and improve the items.

The raw scores were entered in Microsoft Excel to compute the item-level FVI (I-FVI) and scale-level FVI (S-FVI). We applied two methods for calculating S-FVI, namely: i) the average of the I-FVI scores (S-FVI/Ave) and ii) the proportion of items with a score of 3 or 4 by all raters (S-FVI/UA) (9). We recoded the rating of 3 or 4 as 1, and otherwise as 0 as suggested by Yusoff (9) for the computation of FVI indices. The acceptable FVI cut-offs were at least 0.83 (9).

### Internal Structure Validation

The validity evidence based on internal structure in this study was obtained based on the factor structure and reliability (11). We employed EFA to examine the factor structure of the items, evaluating the perceived benefits and barriers to determine the key dimensions of benefits and barriers associated with the flexible work options. Principal axis factoring extraction with Promax rotation method was used to reduce dimensionality because it had less emphasis on multivariate normality and potential correlations between the factors, as they might be conceptually linked (12, 13). To verify the data suitability for EFA, the Kaiser-Meyer-Olkin (KMO) measure of sampling adequacy value should score 0.6 or above and the Bartlett’s test of sphericity value should be significant (14).

Item factor loadings and their significance were assessed to confirm the unidimensionality of the measure. Minimum acceptable factor loading was defined as 0.3 or higher, with a value of 0.5 and above indicating practical significance (12). Ultimately, the factor with at least three items with loadings higher than 0.4 would be retained (15). There were four initially proposed domains for this tool; however, throughout the factor structure evaluation process, the items revealed a three-factor structure. As three domains were identified, the titles were subsequently changed to reflect more appropriate terminologies, namely ‘perceived benefits-workplace management’; ‘perceived benefits-family life balance’ and ‘perceived barriers.’

Following that, the reliability analysis was applied to examine the internal consistency. The internal consistency of the tool was examined using Cronbach’s alpha. In this study, we adopted a minimum threshold of 0.7 for satisfactory internal consistency (12, 16).

### Sample Size Calculation

Using the 10:1 subject to item ratio, the minimum sample size for EFA was 210 respondents (17). The calculation for reliability analysis was based on a conventional choice of level of significance of 95% (type 1 error of 5%), an expected Cronbach alpha of 0.6, which yielded the minimum sample size of 134 (18).

## Results

### Demographic Characteristics

A total of 485 responses were received during the recruitment period. We excluded 105 invalid responses, which contained the exact same answer to all items and 41 unconsented responses. Therefore, 339 usable responses were included in the final analysis. The final sample consisted of 208 nurses (61.4%), 43 doctors (12.7%), 17 administrators (5.0%) and 14 pharmacists (4.1%). The average age of the respondents was 34 years old and most of them were female (81.7%), married (65.5%) and were permanent staff (92.3%). Most respondents reported adopting shift work schedules (55.5%). The characteristics of the respondents are presented in Table 2.

### Assessment of Scale Validity and Reliability

#### i) Response Process Validity

In the response process validation, all items in FWAPB had satisfactory response process indices. We obtained the values of I-FVI, S-FVI/Ave and S-FVI/UA of 1.00 for all items. The finding met the acceptable FVI cut-off of at least 0.83 (9). As the validity evidence was based on response processes obtained, no change in the wording was made in any of the items.

#### ii) Internal Structure

The factor structure of FWAPB was examined in EFA using principal axis factoring extraction with Promax rotation method. KMO value was 0.898 and the Bartlett’s test of sphericity value was significant. Hence EFA was appropriate. In the factor analysis, all items met the minimum acceptable factor loadings of 0.3 (12). However, Factor 4 consisted of two items with factor loadings of < 0.4 (items 3 and 4). Both items had cross-loaded on Factor 1 and Factor 2 with larger loadings, respectively. Table 3 summarises the initial four extracted factors of the 21-item scale.

As Factor 4 contained only two items which had larger loadings on the other factors, namely Factor 1 and Factor 2, we aimed to remove Factor 4 and retain three factors by fixing the number of factors to a three-factor solution. This was desirable to retain the factors with at least three items with loadings higher than 0.4 (15). The final EFA, therefore, consisted of three factors with 21 items. The factors identified explained a total item variance of 56.7% and corresponded to three dimensions, namely perceived benefits-workplace management (Factor 1), perceived benefits-family life balance (Factor 2) and perceived barriers (Factor 3). All 21 items in the final three-factor FWAPB scale met the minimum acceptable factor loadings of 0.3 (12).

In addition, Cronbach’s alpha indicated good internal consistency for each factor and ranged between 0.852 and 0.884. The analysis also showed that Cronbach’s alpha would not increase with item removal. Hence, no item was deleted from the scale and no change made to the items. Table 4 displays the mean, SD, factor loading and Cronbach’s alpha of the final three-factor structure. As there is no amendment to the items, we analysed and explored the perceived benefits and barriers among the healthcare workers and included the result of the main analysis in the subsequent sections of this paper.

### Exploratory Study on the Implementation of Flexible Work Arrangement among Healthcare Workers in Miri Hospital

#### i) Preference and Perceived Feasibility on the Types of FWA

In the current study, we also attained the information on the types of FWA used in the respondents’ current workplace. The respondents may select all FWA options used. Our findings showed that shift work was most commonly used (61.4%), followed by flextime (34.5%), telecommuting (9.4%) and condensed work week (7.7%). The least frequently used FWA option was remote working (3.5%). The respondents preferred flextime and viewed it as the most feasible type of FWA. Meanwhile, remote working was the least preferred and viewed as the least feasible FWA option in their workplace (Table 5).

#### ii) Perceived Benefits and Barriers of FWA

Most of the respondents agreed that FWA helps to improve social distancing among colleagues (mean = 3.65, SD = 0.99) and reduced their exposure to COVID-19 (mean = 3.60, SD = 1.06). Some viewed that FWA helped to reduce work-related stress levels (mean = 3.59, SD = 0.98).

From the perspective of the perceived barriers, most respondents disagreed that FWA had negative effects on their personal well-being (mean = 2.49, SD = 0.97) and their family well-being (mean = 2.56, SD = 0.99). In addition, the respondents tended to disagree that FWA reduced their work productivity. The respondents perceived that the greatest barrier in FWA was the distraction associated with working out of the office (mean = 2.86, SD = 1.00).

#### iii) Readiness to Implement FWA

The mean scores for readiness statements ranged from 3.42 to 3.50 and 44.5% of the respondents viewed that they would opt for FWA at their workplace (mean = 3.47, SD = 0.99) whereas 45.7% of the respondents were confident to adapt to FWA and 44.0% of them believed that Miri Hospital was ready to implement FWA (Table 6).

## Discussion

### Assessment of Scale Validity and Reliability

The current study has developed the FWAPB, which can be used to assess perceived benefits and barriers of FWA. It is commonly understood that validity and reliability relate to the interpretation of psychometric instruments (11). A unitary concept ‘construct validity’ has gradually replaced prior distinctions of face, content, and criterion validity. According to Cook and Beckman (11), there are several sources of evidence to support the validity argument. In this study, we sought for validity evidence from the response process and internal structure to support the construct validity of the scale. Response process validity, which is also known as face validity (9), is the thought processes of test respondents as they respond to the assessment tool (19). In this study, all items had satisfactory response process indices, subsequently no changes in the wording were made.

On the other hand, reliability and factor analysis are considered evidence of the internal structure validity (11). In this study, factor analysis of the FWAPB discovered three dimensions of perceived benefits and barriers of FWA which are: i) perceived benefits-workplace management; ii) perceived benefits-family life balance and iii) perceived barriers. In addition, the scale was found to have good internal consistency with Cronbach’s alpha for each of the factors (0.852–0.884). As the deletion of any items would not escalate the alpha coefficient, retaining all items of FWAPB was reasonable.

It is known that in self-administered surveys, it is common to observe insufficient effort and careless responding (20). Due to the concerns over the threat and distortion that invalid responses might have on the distinction of theoretically distinct factors (21), we attempted to eliminate the biases arising from the respondents who paid little attention and put little effort in providing thoughtful responses. In this study, a significant number of respondents gave exactly the same response to all items. Thus, we filtered out these responses from the final analysis. However, an EFA which incorporated the excluded responses was subsequently performed to confirm the properties in the complete samples (Appendix 1) and compare them to the clean samples. The analysis revealed similar dimensionality, factor structure and data reliability in both measurement models. Hence, the overall scale of FWAPB as determined by psychometric testing can be considered valid and reliable.

### Exploratory Study on the Implementation of Flexible Work Arrangement among Healthcare Workers in Miri Hospital

Identifying the underpinned factors related to the perceived benefits and barriers of the implementation of FWA in a hospital setting may help to identify employees’ concerns in adopting FWA, determine the employees’ participation, and hence may facilitate the implementation of FWA, especially among healthcare workers. In this study, most respondents perceived that FWA is beneficial in the risk management of COVID-19 in the workplace. The majority of the respondents were concerned in improving social distancing among colleagues and to reduce exposure to COVID-19. A study showed that there was a statistically significant association in social distancing measures with a decrease in the COVID-19 case growth rate (22). In addition to the workplace management perspective, FWA was perceived to bring benefits for healthcare workers in terms of family life balance. This is supported by previous studies which found that FWA is associated with positive outcomes for employees (23, 24).

FWAs were predominantly applied in the professional service and IT sector (25). Previous studies on FWAs focused on groups such as accounting professionals, IT, banking, insurance, manufacturing, educational and research (7, 25, 26). Literature remains limited especially addressing the implementation of FWA among public healthcare workers and particularly during the pandemic. Due to the 24-h operation in a hospital setting, shift work is routinely practised. Nevertheless, the current study showed that flextime is the most preferred option. As compared to other flexible work options, flextime offers flexibility in the timing of work. A previous study reported that there is a positive correlation between flextime with work-family balance and job satisfaction (27). Meanwhile, remote working is the least preferred and thought to be the least feasible option. Nonetheless, the use and advance of technology has enabled and catalysed the use of telecommuting, teleconsultation and virtual continuation of medical education. There is growing evidence that the conventional consultation could possibly be replaced by emerging technology-driven methods such as tele-rehabilitation-based consultation. In physiotherapy, for instance, the use of tele-rehabilitation in musculoskeletal pain is reported to be feasible (28). Another study conducted among stroke patients revealed improvement in their quality of life and depression through tele-rehabilitation (29).

While some authors argued that public sector employees were more likely to perceive greater benefits and exhibited fewer concerns (8). One study found that individuals with a strong preference for integrating their work and non-work roles were signiﬁcantly more attracted to FWAs (30). Readiness to adopt FWA depends on the person-environment fit theory (family, work and community) (31). From the readiness statements, almost half of our respondents were positive towards the implementation of FWAs, whereas about ten per cent of the respondents were not ready. However, we also observed that a high percentage of respondents remained neutral. This could be possibly due to the short implementation period of various FWAs in our setting.

Therefore, a one-size-fit-all approach is not workable as different individuals will choose different approaches of FWAs to meet their needs (31). As the pandemic led to consequences such as school closures and home-based learning, FWAs could be perceived more valuable to working mothers and dual-career couples, which may increase their readiness to adopt the options. Nevertheless, research conducted to explore the success and failure of FWAs in healthcare organisations revealed that not all healthcare organisations successfully implement flexible schedules (32, 33). There are obstacles to overcome to facilitate long-term positive outcomes. When patient demand is high, the work arrangement can lead to stressful conditions whilst burnout symptoms are likely to happen (34). Investigation among registered nurses showed that high patient load is related to higher restraint use and more patient deaths (35). Another study in Australia linked the high patient demand with low healthcare quality indicators. It identified an increase in patient mortality during observed periods of overcrowding within the emergency department (36). However, some stated that managing the scheduling of work to best support employee work-life balance is stressful (37). The successful implementation of FWAs requires commitment from both employers and employees, hence this could be challenging.

A key strength of the present study was the sufficient sample recruited. In addition, the sample provided a comprehensive representation of the hospital population. However, there are several limitations in this study. Firstly, this is a single-centred study. Therefore, the result may not be generalisable to a population with different characteristics. Secondly, female healthcare members contributed disproportionately to the respondent data set, as compared to the male members. This is similar to some of the past studies, which asserted that a larger percentage of female respondents would return surveys than their male counterparts (38, 39). This may suggest an overall response bias. Moreover, the current study did not address the difference in perception and readiness among employees with different characteristics. Thus, the researchers recommend that future research may explore the effect of characteristics, such as gender, generation group and marital status, on their opinions on such arrangements using subgroup analysis. Other analyses, such as latent construct analysis, may also be useful to explore the relationship between the variables. Moreover, future researchers may conduct a qualitative study to discover the obstacles among those who are not ready to implement FWAs.

## Conclusion

In conclusion, the FWAPB was found to have three factors and satisfies the response process validity, construct validity and reliability. Hence, it is a valid and reliable tool. Almost half of the respondents were positive towards FWA implementation. Yet, a high proportion of them remained neutral on its implementation. These findings contribute to the understanding of concerns that must be addressed to increase the readiness and acceptance of such arrangements. It is recommended that the management should look into different models of FWAs to suit different groups of healthcare workers.

## Figures and Tables

**Table 1:** The initial items and preliminary domains of FWAPB scale

Domains	Items
Work-family balance	FWA has negative effects on my family well-being (R) FWA is important for me in attending to family responsibilities FWA is important for me in attending family events FWA makes it difficult for me to attend to my family needs (R) FWA allows me to spend time with my family
Work-life benefits	FWA helps to reduce work-related stress level FWA allows me to spend time in my favourite personal activity FWA enable me to deal with my other personal commitments FWA has negative effects on my personal well-being (R) FWA disrupts too much of my life (R)
Workplace management	FWA reduces my workload FWA helps in job sharing among colleagues FWA improves work efficiency FWA causes me to miss important work events (e.g. meetings, training sessions) (R) FWA makes me feel disconnected from the workplace (R) FWA reduces my work productivity (R) FWA causes less commitment to my work role (R) I am easily distracted if working out of the office (e.g. from home) (R)
COVID-19 risk management	FWA helps to reduce the patient overcrowding FWA helps in improving the social distancing among colleagues FWA reduces my exposure to COVID-19

Note: (R) = refers to the negatively worded statements

**Table 2:** Demographic characteristics of respondents (*N* = 339)

Variables		Mean (SD)	*n* (%)
Gender	Male		62 (18.3)
	Female		277 (81.7)
Age (years old)		34.20 (7.06)	
Ethnicity	Malay		92 (27.1)
	Chinese		61 (18.0)
	Indian		8 (2.4)
	Native of Sarawak		178 (52.5)
Marital status	Single		111 (32.7)
	Married		222 (65.5)
	Divorced		5 (1.5)
	Widowed		1 (0.3)
Number of dependent under care	0		85 (25.1)
	1		40 (11.8)
	2		75 (22.1)
	3		53 (15.6)
	4		47 (13.9)
	≥ 5		39 (11.5)
Type of service scheme	Support group II (Grade 1–16)		15 (4.4)
	Support group I (Grade 17–40)		260 (76.7)
	Administrative, Professional (Grade 41–56)		64 (18.9)
Staff position	Nurse		208 (61.4)
	Doctor		43 (12.7)
	Medical assistant		10 (2.9)
	Pharmacist		14 (4.1)
	Physiotherapist		13 (3.8)
	Administration/management		17 (5.0)
	Health care assistant		13 (3.8)
	Others		21 (6.2)
Number of years in service		9.76 (6.98)	
Type of appointment	Permanent		313 (92.3)
	Contract		26 (7.7)
Current working system	Office hours		129 (38.1)
	Shift system		188 (55.5)
	Combination of office hours, shift and on call		22 (6.5)

**Table 3:** Initial factor analysis for the FWAPB scale (*N* = 339)

No.	Items	Factor 1	Factor 2	Factor 3	Factor 4
1	FWA has negative effects on my family well-being	0.412			
2	FWA helps to reduce work-related stress level		0.577		
3	FWA reduces my work productivity	**0.599**			0.321
4	FWA reduces my workload		**0.576**		−0.363
5	FWA helps to reduce the patient overcrowding		0.647		
6	FWA is important for me in attending to family responsibilities			0.625	
7	FWA allows me to spend time in my favourite personal activity			0.831	
8	FWA causes less commitment to my work role	0.530			
9	FWA helps in job sharing among colleagues		0.718		
10	FWA helps in improving the social distancing among colleagues		0.827		
11	FWA is important for me in attending family events			0.903	
12	FWA enable me to deal with my other personal commitments			0.876	
13	I am easily distracted if working out of the office	0.493			
14	FWA improves work efficiency		0.558		
15	FWA reduces my exposure to COVID-19		0.793		
16	FWA makes it difficult for me to attend to my family needs	0.709			
17	FWA has negative effects on my personal well-being	0.752			
18	FWA causes me to miss important work events	0.758			
19	FWA makes me feel disconnected from the workplace	0.663			
20	FWA allows me to spend time with my family			0.500	
21	FWA disrupts too much of my life	0.704			

Note: Underlined values indicate a double loading on different factors. Loadings highlighted in bold indicate the factor on which the item placed

**Table 4:** Mean, SD, factor loading and Cronbach’s alpha coefficient of the items (a three-factor structure) (*N* = 339)

No.	Items	Mean (SD)	Floor effect a (%)	Ceiling effect b (%)	Factor 1	Factor 2	Factor 3	Cronbach’s alpha
2	FWA helps to reduce work-related stress level	3.59 (0.98)	2.7	18.0	0.594			0.852
4	FWA reduces my workload	3.07 (1.01)	7.7	8.0	0.596			
5	FWA helps to reduce the patient overcrowding	3.14 (1.10)	8.6	10.6	0.662			
9	FWA helps in job sharing among colleagues	3.43 (0.90)	3.5	10.0	0.722			
10	FWA helps in improving the social distancing among colleagues	3.65 (0.99)	3.8	19.2	0.804			
14	FWA improves work efficiency	3.46 (0.88)	3.5	11.2	0.556			
15	FWA reduces my exposure to COVID-19	3.60 (1.06)	5.3	21.8	0.754			

6	FWA is important for me in attending to family responsibilities	3.57 (0.98)	3.8	16.2		0.620		0.884
7	FWA allows me to spend time in my favourite personal activity	3.53 (0.98)	4.1	15.6		0.830		
11	FWA is important for me in attending family events	3.44 (1.03)	4.7	15.3		0.902		
12	FWA enable me to deal with my other personal commitments	3.49 (1.00)	4.7	14.2		0.878		
20	FWA allows me to spend time with my family	3.50 (0.96)	2.9	15.3		0.489		

1	FWA has negative effects on my family well-being	2.56 (0.99)	16.2	2.9			0.476	0.854
3	FWA reduces my work productivity	2.66 (0.93)	10.9	2.1			0.666	
8	FWA causes less commitment to my work role	2.73 (0.99)	11.2	4.1			0.579	
13	I am easily distracted if working out of the office	2.86 (1.00)	11.2	3.8			0.522	
16	FWA makes it difficult for me to attend to my family needs	2.76 (0.99)	10.6	4.7			0.712	
17	FWA has negative effects on my personal well-being	2.49 (0.97)	16.5	3.5			0.768	
18	FWA causes me to miss important work events	2.71 (0.97)	10.3	4.1			0.713	
19	FWA makes me feel disconnected from the workplace	2.65 (0.93)	9.7	2.9			0.602	
21	FWA disrupts too much of my life	2.58 (1.00)	16.8	3.5			0.698	

Notes:

a Floor score = 1;

b ceiling score = 5.

Factor 1 refers to perceived benefits-workplace management; Factor 2 refers to perceived benefits-family life balance; and Factor 3 refers to perceived barriers

**Table 5:** Preference and perceived feasibility on the types of FWA (*N* = 339)

	Types of FWA	*n* (%)					Mean (SD)

		1	2	3	4	5	
Preference	Telecommuting	74 (21.8)	65 (19.2)	82 (24.2)	46 (13.6)	72 (21.2)	2.93 (1.43)
	Remote working	43 (12.7)	48 (14.2)	88 (26.0)	55 (16.2)	105 (31.0)	3.39 (1.38)
	Condensed work week	39 (11.5)	72 (21.2)	97 (28.6)	41 (12.1)	90 (26.5)	3.21 (1.35)
	Flextime	120 (35.4)	93 (27.4)	72 (21.2)	25 (7.4)	29 (8.6)	2.26 (1.25)
	Shift work	114 (33.6)	70 (20.6)	75 (22.1)	22 (6.5)	58 (17.1)	2.53 (1.44)
Feasibility	Telecommuting	53 (15.6)	68 (20.1)	80 (23.6)	59 (17.4)	79 (23.3)	3.13 (1.39)
	Remote working	29 (8.6)	56 (16.5)	90 (26.5)	60 (17.7)	104 (30.7)	3.45 (1.31)
	Condensed work week	39 (11.5)	67 (19.8)	108 (31.9)	55 (16.2)	70 (20.6)	3.15 (1.28)
	Flextime	116 (34.2)	102 (30.1)	68 (20.1)	35 (10.3)	18 (5.3)	2.22 (1.18)
	Shift work	123 (36.3)	75 (22.1)	65 (19.2)	35 (10.3)	41 (12.1)	2.40 (1.38)

Notes: For preference, 1 indicates the most preferred FWA type; 5 indicates the least preferred type. For feasibility, 1 indicates the most feasible FWA type; 5 indicates the least feasible one

**Table 6:** Readiness to implement FWA (*N* = 339)

Items	*n* (%)					Mean (SD)

	1	2	3	4	5	
I will opt for FWA	17 (5.0)	15 (4.4)	156 (46.0)	93 (27.4)	58 (17.1)	3.47 (0.99)
I am confident I am able to adapt to FWA	12 (3.5)	13 (3.8)	159 (46.9)	104 (30.7)	51 (15.0)	3.50 (0.92)
Miri Hospital is ready to implement FWA	14 (4.1)	27 (8.0)	149 (44.0)	102 (30.1)	47 (13.9)	3.42 (0.96)

Note: 1 = strongly disagree; 2 = disagree; 3 = neutral; 4 = agree; 5 = strongly agree

## Appendix 1

Mean, standard deviation, factor loading and Cronbach alpha’s coefficient of the items (a three-factor structure) of the complete samples (*N* = 444)

**Table:**

No.	Items	Mean (SD)	Floor effect^a^ (%)	Ceiling effect^b^ (%)	Factor 1	Factor 2	Factor 3	Cronbach’s alpha
2	FWA helps to reduce work-related stress level	3.45 (0.92)	2.7	13.7	0.658			0.875
4	FWA reduces my workload	3.05 (1.01)	6.5	6.1	0.605			
5	FWA helps to reduce the patient overcrowding	3.10 (0.99)	7.2	8.1	0.711			
9	FWA helps in job sharing among colleagues	3.33 (0.84)	3.4	7.7	0.741			
10	FWA helps in improving the social distancing among colleagues	3.49 (0.94)	3.6	14.6	0.821			
14	FWA improves work efficiency	3.35 (0.82)	3.4	8.6	0.601			
15	FWA reduces my exposure to COVID-19	3.46 (0.99)	4.7	16.7	0.778			

6	FWA is important for me in attending to family responsibilities	3.43 (0.92)	3.3	11.3		0.619		0.902
7	FWA allows me to spend time in my favourite personal activity	3.41 (0.92)	3.8	11.9		0.824		
11	FWA is important for me in attending family events	3.33 (0.94)	3.9	10.7		0.911		
12	FWA enable me to deal with my other personal commitments	3.37 (0.92)	4.3	10.8		0.886		
20	FWA allows me to spend time with my family	3.38 (0.89)	2.9	11.7		0.499		

1	FWA has negative effects on my family well-being	3.34 (0.91)	12.0	2.1			0.564	0.879
3	FWA reduces my work productivity	3.26 (0.86)	9.0	1.6			0.704	
8	FWA causes less commitment to my work role	3.21 (0.90)	9.2	3.2			0.582	
13	I am easily distracted if working out of the office	3.11 (0.90)	9.2	2.9			0.553	
16	FWA makes it difficult for me to attend to my family needs	3.19 (0.90)	8.8	3.6			0.744	
17	FWA has negative effects on my personal well-being	3.39 (0.91)	13.3	2.7			0.796	
18	FWA causes me to miss important work events	3.23 (0.89)	8.6	3.2			0.744	
19	FWA makes me feel disconnected from the workplace	3.27 (0.86)	8.1	2.3			0.649	
21	FWA disrupts too much of my life	3.33 (0.92)	13.5	2.7			0.744	

Note:

a Floor score = 1;

b ceiling score = 5.

Factor 1 refers to perceived benefits-workplace management; Factor 2 refers to perceived benefits-family life balance; and Factor 3 refers to perceived barriers

## References

[1] Lewis S (2003). Flexible working arrangements: Implementation, outcomes, and management. Int Rev Ind Organ Psychol.

[2] Kossek E, Hammer L, Thompson R, Burke L (2014). Leveraging workplace flexibility: fostering engagement and productivity. SHRM foundation’s effective practice guidelines series.

[3] Shagvaliyeva S, Yazdanifard R (2014). Impact of flexible working hours on work-life balance. Am J Ind Business Manag.

[4] Albion MJ (2004). A Measure of attitudes towards flexible work options. Austr J Manag.

[5] Queensland Government Types of flexible working arrangements (updated 19 August 2020).

[6] Isa SM, Musa M, Gee CA (2016). The female employees’ readiness on flexible working arrangements in Malaysia.

[7] Charron KF, Lowe DJ (2005). Factors that affect accountant’s perceptions of alternative work arrangements. Accounting Forum.

[8] Giannikis SK, Mihail DM (2011). Flexible work arrangements in Greece: a study of employee perceptions. Int J Human Resource Manag.

[9] Yusoff MSB (2019). ABC of response process validation and face validity index calculation. Edu Med J.

[10] American Educational Research Association (2014). Standards for educational and psychological testing. 2014 ed.

[11] Cook DA, Beckman TJ (2006). Current concepts in validity and reliability for psychometric instruments: theory and application. Am J Med.

[12] Hair JF, Anderson RE, Babin BJ, Black WC (2010). Multivariate data analysis: a global perspective.

[13] Yong AG, Pearce S (2013). A Beginner’s guide to factor analysis: focusing on exploratory factor analysis. Tutor Quant Methods Psychol.

[14] Pallant J (2010). Surviving SPSS manual: a step by step guide to data analysis using SPSS.

[15] Samuels P Advice on exploratory factor analysis [Internet].

[16] Bland JM, Altman DG (1997). Statistical notes: Cronbach’s Alpha. BMJ.

[17] Costello AB, Osborne J (2005). Best practices in exploratory factor analysis: four recommendations for getting the most from your analysis. Practical Assessment, Research, and Evaluation.

[18] Arifin WN (2017). Sample size calculator (version 2.0) [Spreadsheet file].

[19] Holden RR, Weiner IB, Craighead WE (2010). Face validity. The Corsini encyclopedia of psychology.

[20] Arias VB, Garrido LE, Jenaro C, Martínez-Molina A, Arias B (2020). A little garbage in, lots of garbage out: assessing the impact of careless responding in personality survey data. Behav Res Methods.

[21] Kam CCS (2019). Careless responding threatens factorial analytic results and construct validity of personality measure. Front Psychol.

[22] Siedner MJ, Harling G, Reynolds Z, Gilbert RF, Haneuse S, Venkataramani AS (2020). Social distancing to slow the US COVID-19 epidemic: longitudinal pretest–posttest comparison group study. PLoS Med.

[23] Baltes BB, Briggs TE, Huff JW, Wright JA, Neuman GA (1999). Flexible and compressed workweek schedules: a meta-analysis of their effects on work-related criteria. J Appl Psychol.

[24] Kossek EE, Michel JS (2011). Flexible work schedules. APA handbook of industrial and organizational psychology, 1: building and developing the organization APA handbooks in psychology.

[25] Mamatha M, Lakshmi B (2020). Influence of employees’ perception on the use of flexible work arrangements. Int J Sci Technol Res.

[26] Laundon M, Williams P (2018). Flexible work: barrier to benefits?. Financial Plan Res J.

[27] Rocereto JF, Gupta SF, Mosca JB (2011). The role of flextime appeal on family and work outcomes among active and non-active flextime users: a between groups and within groups analysis. J Bus Econ Res.

[28] Turolla A, Rossettini G, Viceconti A, Palese A, Geri T (2020). Musculoskeletal physical therapy during the COVID-19 pandemic: is telerehabilitation the answer?. Phys Ther.

[29] Linder SM, Rosenfeldt AB, Bay RC, Sahu K, Wolf SL, Alberts JL (2015). Improving quality of life and depression after stroke through telerehabilitation. Am J Occup Ther.

[30] Thompson RJ, Payne SC, Taylor AB (2015). Applicant attraction to flexible work arrangements: separating the influence of flextime and flexplace. J Occup Organ Psychol.

[31] Isaa SM, Musab M, Geec CA (2016). The female employees’ readiness on flexible working arrangements in Malaysia. Gen Stu.

[32] Pryce J, Albertsen K, Nielsen K (2006). Evaluation of an open-rota system in a Danish psychiatric hospital: a mechanism for improving job satisfaction and work-life balance. J Nurs Manag.

[33] Wallace L-A, Pierson S (2008). A case study: the initiative to improve RN scheduling at Hamilton Health Sciences. Nurs Leadersh.

[34] Sherman AC, Edwards D, Simonton S, Mehta P (2006). Caregiver stress and burnout in an oncology unit. Palliat Support Care.

[35] Unruh L, Joseph L, Strickland M (2007). Nurse absenteeism and workload: negative effect on restraint use, incident reports and mortality. J Adv Nurs.

[36] Richardson DB (2006). Increase in patient mortality at 10 days associated with emergency department overcrowding. Med J Aust.

[37] Skinner N, Van Dijk P, Elton J, Auer J (2011). An in-depth study of Australian nurses’ and midwives’ work-life interaction. Asia Pac J Hum Resour.

[38] Smith WG (2008). Does gender influence online survey participation? A record-linkage analysis of university faculty online survey response behavior.

[39] Yetter G, Capaccioli K (2010). Differences in responses to Web and paper surveys among school professionals. Behav Res Methods.

